# Severe Potential Drug-Drug Interactions and the Increased Length of Stay of Children in Intensive Care Unit

**DOI:** 10.3389/fphar.2020.555407

**Published:** 2020-12-03

**Authors:** Elisangela da Costa Lima, Barbara Dias Camarinha, Nathalia Cristina Ferreira Bezerra, Anderson Gonçalves Panisset, Raquel Belmino de Souza, Marcus Tolentino Silva, Luciane Cruz Lopes

**Affiliations:** ^1^ Pharmacy School, Rio de Janeiro Federal University, Rio de Janeiro, Brazil; ^2^ Instituto de Puericultura e Pediatria Martagão Gesteira, Rio de Janeiro Federal University, Rio de Janeiro, Brazil; ^3^ Graduate Course of Pharmaceutical Science, Universidade de Sorocaba, Sorocaba, Brazil

**Keywords:** child, drug interactions, intensive care units, pediatric, drug utilization review, hospital stay

## Abstract

Children are exposed to drug-drug interactions (DDI) risks due to their organism’s complexity and the need for several medicines prescriptions in pediatric intensive care units (PICU). This study aimed to assess the prevalence of potential DDIs in a Brazilian PICU. We carried out a cross-sectional study at a pediatric teaching hospital from Rio de Janeiro (Brazil) over one year. Potential DDIs (pDDIs) between prescribed medicines for hospitalized children in PICU (*n* = 143) were analyzed according to severity using Micromedex^®^. Sex, age group, number of drugs prescribed, vasoactive amines use (a proxy of clinical complexity), and the PICU length of stay were summarized using descriptive statistics. Association between the PICU length stay, and variables sex, age, clinical condition complexity, number of drugs prescribed, and severity of pDDI were examined by univariate and multiple linear regression. Seventy percent of patients aged three days to 14 years old were exposed at least one potential DDIs during PICU stay. Two hundred eighty-four different types of pDDIs were identified, occurring 1,123 times. Nervous system drugs were implicated in 55% of the interactions, and fentanyl (10%) was most involving in pDDIs. Most pDDIs were classified as higher severity (56.2%), with reasonable documentation (64.6%) and unspecified onset time (63.8%). Worse clinical condition, ten or more drugs prescribed, and most severe pDDIs were associated with a longer PICU length of stay. Multiple linear regression analysis showed an increase of 9.83 days (95% confidence interval: 3.61–16.05; *p* = 0.002) in the PICU length of stay in children with major or contraindicated pDDIs. The results of this research may support the monitoring and prevention of pDDIs related to adverse events in children in intensive care and the design and conduction of new studies.

## Introduction

Adverse drug events (ADE) are among the leading causes of increased morbidity, mortality, and health costs (Dai et al., 2016). Children admitted to critical care units are more exposed to pharmacotherapy damage risk due to several phases and changes in their development, different response mechanisms to harms, and multiple medicines prescription (Silva et al., 2013). The hepatic and urinary systems’ maturation is slow, which means less expression or even lack of cytochrome P450 enzymes (CYP1A2, CYP2C9, CYP2C19, and CYP2D6 isoenzymes), decreased renal blood flow, glomerular filtration, and tubular function, chiefly until the age of three-years-old (Fernandez et al., 2011). These variations may affect the absorption, distribution, metabolism, and elimination of drugs in children, increasing the risk of toxicity (Fernandez et al., 2011).

The combination of several drugs and the occurrence of drug interactions in pediatric intensive care units (PICU) is frequently unavoidable and needed during the patient stabilization process, diagnosis, and specific treatment but increases the risk of toxicity and can reduce therapeutic’s efficacy (Brunton et al., 2010; Silva, 2012; Dai et al., 2016). Drug-drug interaction (DDI) is defined as a clinical event in which one drug’s effect is significantly modified by the presence of another previously or concurrently administered drug. Potential DDI (pDDI) refers to the possibility, in theory, of one drug physiologically altering the pharmacological effects of another drug, concomitantly prescribed (Brunton et al., 2010).

DDIs may benefit clinical management when one drug is used to optimize another drug’s action, as ascorbic acid and non-heme iron in concomitant use, for example, (Brunton et al., 2010; Queiroz et al., 2014). However, undesirable DDIs are related to ADE and increased length of hospital stay (Khan, 2013; Alvim et al., 2015; Fitzmaurice et al., 2019).

Knowledge about pDDIs in child health care may contribute to monitoring and minimizing ADE and treatment failures. The literature is scarce on pediatric pharmacoepidemiological studies, especially in developing countries (Osokugu et al., 2016), which motivated the investigation of the prevalence of pDDI in a PICU in a Brazilian teaching hospital.

## Methods

### Design and Setting

A cross-sectional study was conducted, and data were collected over one year (May 2014 and April 2015) at a pediatric teaching hospital located in Rio de Janeiro (Brazil) and integrated into the public health system. In addition to outpatient care in general pediatrics, the hospital studied had a pediatric emergency service, oncohematology, and surgical hospitalization for medium and high-complexity care. PICU’s installed capacity was ten hospital beds, of which six were pediatric beds and four neonatal surgical beds, and one isolation room.

### Eligibility Criteria

All patients aged 0–17 years old admitted to the PICU who stayed for more than 24 h and were administered at least two medicines during hospitalization were included. No exclusion criteria were applied.

### Variables Collected and Other Measures

Data was collected from 1) medical records: sex, age on the first day of hospitalization, the length of PICU stays in days, cause of admission, and severity of illness; 2) daily records of the prescriptions using the electronic hospital management system: name, dose and route of administration; and 3) pharmacotherapeutic plans prepared by the clinical pharmacist: pDDI reported and any information possibly incomplete in data sources mentioned above.

Children were classified by age group as neonates (0–28 days old); infants (29 days–11 months old); toddlers (1 year–2 years and 11 months old); preschoolers (3–5 years and 11 months old); middle childhood (6–11 years and 11 months old) and teenagers (12–16 years old) (Osokugu et al., 2016). Readmissions of the same patient were considered as new cases.

Patient’s diagnoses (main hospitalization cause) were classified according to the International Classification of Diseases (ICD10). None score that predicted morbidity or mortality of children was used in the investigated PICU. The prescription at least one vasoactive amine among those commonly used in PICU (epinephrine, norepinephrine, dobutamine, and nitroprusside by parenteral route) was the variable considered as a *proxy* for patient’s clinical condition. These drugs were used to restore tissue perfusion in hemodynamically unstable patients by the drastic and widespread reduction of effective oxygen and other nutrient delivery to tissues, leading to cell damage and multiple organ failure (Guimarães et al., 2008; Paediatric Intensive Care Pharmacist’s Special Interest Group Neonatal and Paediatric Pharmacists Group, 2011). The need of vasoactive amines was described as a clinical signal of seriousness and worse prognostic in this study (Roque et al., 2016; Pollack et al., 2018).

All prescribed drugs were classified according to the Anatomical Therapeutic Chemical (ATC) classification system recommended by the World Health Organization. The number of concomitant drugs prescribed was stratified in three groups 1): two to four drugs; 2) five to nine drugs (polypharmacy) and 3) ten or more drugs (excessive polypharmacy) (Dai et al., 2016). Drugs prescribed *Pro re nata* (if needed) were excluded. Topical medications, electrolyte solutions, parenteral and enteral nutrition also were not considered in the analysis.

Each potential DDIs identified were characterized using the *Thomson Micromedex* ^®^ software; since it is a database that gathers a larger number of drug monographs, it is available at the studied hospital and is easily accessible online. They were classified according to the level of scientific evidence documentation (excellent, good, reasonable) about pDDI, severity (minor, moderate, major, and contraindicated), and the onset of action (fast, slow, unknown). Minor pDDI may generate limited clinical outcomes, including an increase in the frequency or seriousness of adverse drug reactions and therapeutics changes. Moderate pDDi may result in aggravation of the children’s condition and require an adjustment in therapy. Major pDDI may be life-threatening and need medical intervention to minimize or prevent serious adverse drug reactions (Micromedex Healthcare Series, 2019).

### Statistical Analysis

We carried out a descriptive analysis of cases including sex, age group, clinical condition (vasoactive amines use), number of drugs prescribed (two to four; five to nine; ten or more), and PICU length of stay (mean as a cut-off point) by the occurrence of pDDI severity (minor and moderate; major and contraindicated) or not.

Association between the PICU length of stay and sex, age group, clinical condition, number of drugs prescribed, and severity of pDDI were verified by linear regression.

Multivariate linear regression, adjusted by sex, age group and clinical condition was performed to assess whether the length of stay in a PICU was influenced by severity of pDDI. All the analyses were performed using Stata (v.14.2), with a calculation of 95% confidence intervals (95%CI). Statistically significant was considered if p < 0.005.

### Ethical Issues

This observational study was approved by the Instituto de Puericultura e Pediatria Martagão Gesteira Research Ethics Committee (REC) (CAAE 52065415.5.0000.5264/Number of reference: 1,451,562).

Retrospective secondary data was collected without any interaction between researchers and children or parents. Personal information of the participants was kept blinded to investigators. Waiver of parental permission (Written informed consent from the participant’s legal guardian) was requested and authorized by REC.

## Results

### Characterization of the Patients and the Pharmacotherapeutic Profile

**FIGURE 1:**
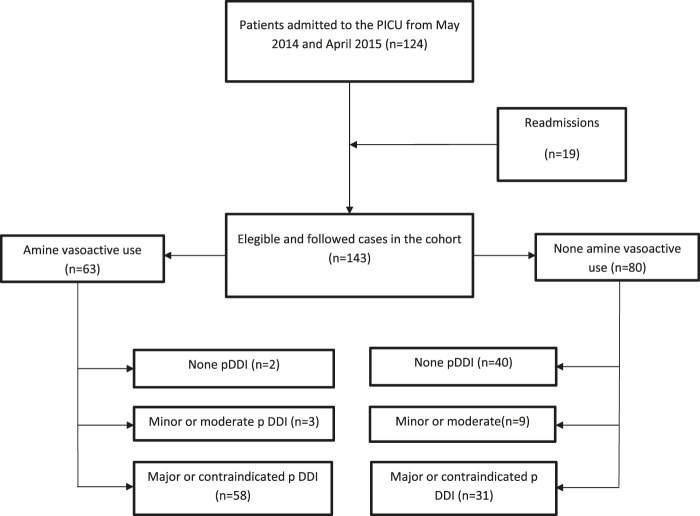
Flow chart of followed cases in the cohort study (May 2014–April 2015).

There were 124 children admitted (for more than 24 h) to the PICU during the investigation period, and they were included. Nineteen were readmitted and included twice in the study, totaling 143 cases analyzed (Figure 1). Their age ranged from three days of life to 14 years old (median = 11 months; mean = 2.6 years; standard deviation ±3.9 years) (Table 1). The distribution of male and female patients was balanced (Table 1). No statistically significant difference was found between the sex and the age of the patients.

**TABLE 1:** Children’s profile distribution by the most severe pDDI observed (Teaching Hospital PICU, Rio de Janeiro, Brazil).

Characterization	pDDI observed			Total	
	None	Minor or moderate	Major or contraindicated	*N*	%
Sex					
Male	23	4	46	73	51
Female	19	8	43	70	49
Clinical condition (vasoactive amines use)					
Yes	2	3	58	63	44
No	40	9	31	80	56
Age group (mean = 2.6 ± 3.9)					
Baby (0–11 months)	26	5	42	73	51
Toddler (12–23 months)	3	4	4	11	8
Early childhood (2–5 years)	6	1	23	30	21
Middle childhood (6–11 years)	5	1	16	22	15
Early adolescence (12–17 years)	2	1	4	7	5
Total number of drugs prescribed (mean = 13.4 ± 8.3)					
2–4	15	0	1	16	11
5–9	24	7	10	41	29
10 or more	3	5	78	86	60
PICU length of stay (mean = 11.3 ± 15.5)					
Until 11 days	41	9	50	100	70
12 or more days	1	3	39	43	30
**Total**	**42**	**12**	**89**	**143**	**100**

pDDI, potential drug-drug interaction; PICU, pediatric intensive care unit.

Most children had respiratory (29.4%), infectious and parasitic (25.9%), or digestive system (9.8%) diseases as the principal diagnosis. During the course of 63 hospitalizations (44%), the use of vasoactive amines was required, indicating more complex clinical cases. The PICU length of stay ranged from one to 113 days, with a mean length of 11.3 days (standard deviation ±15.5). In 43 cases (35%), the length of stay was longer than the mean.

During the period, 1,916 drug prescriptions (with 149 different active ingredients) for 143 children were analyzed (mean = 13.4 ± 8.3).

The number of medicines prescribed per patient ranged from two to 46 medications (median = 12). Most of the patients (60%) used more than ten different medications. Antibacterial for systemic use (19.31%) and analgesic drugs (8.56%) were the most prescribed anatomical subgroups. Table 2 shows the medicines more frequently observed in prescriptions.

**TABLE 2:** Distribution of the most frequently drugs prescribed (Teaching Hospital PICU, Rio de Janeiro, Brazil).

Drugs	ATC Classification	Total	
		*N*	%
Metamizole	N02BB02	130	91
Ranitidine	A02BA02	112	78
Fentanyl	N02AB03	71	50
Midazolam	N05CD08	70	49
Dexamethasone	H02AB02	67	47
Vancomycin	J01XA01	67	47
Cefepime	J01DE01	54	38
Salbutamol	R03CC02	53	37
Methylprednisolone	H02AB04	50	35
Dobutamine	C01CA07	47	33

ATC, Anatomical Therapeutic Chemical.

### Potential Drug-Drug Interactions Frequency

284 different pDDIs prescribed 1,123 occasions were identified for 101 (70.6%) children. Prescriptions of 42 (30.4%) children did not have drugs that interacted with each other throughout the treatment at PICU. The mean of pDDIs observed in prescriptions by total hospitalizations in the period was 7.85 (±0.08).

Fourteen pDDIs were classified as contraindicated, 631 had higher severity, 425, moderate severity, and 53, lower severity. Most of the pDDIs had a reasonable level of evidence (64.6%) and the unspecified onset of action (63.8%).

**FIGURE 2:**
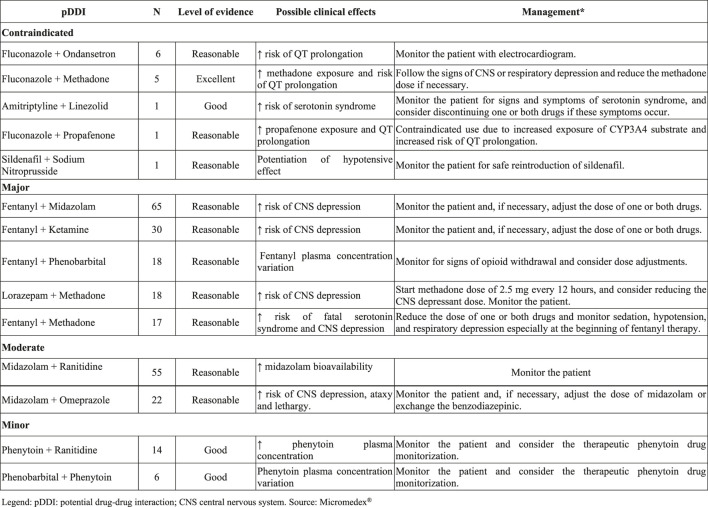
Description of the five most frequently contraindicated and major pDDIs (Teaching Hospital PICU, Rio de Janeiro, Brazil–May/2014–April/2015, *n* = 1,123). *Source as: Micromedex Healthcare Series 2019.

Drugs more involving in pDDI were classified in groups N (Nervous system, 55%), J (Antiinfectives for systemic use, 16%), and A (Alimentary tract and metabolism, 8%) according to ATC code. The most prevalent drugs were phenytoin (5.6%), fentanyl (4.6%), methadone (4.6%), phenobarbital (3.9%), morphine (3.7%), fluconazole (3.3%), cyclosporine (3.2%), clarithromycin (3%), midazolam (2.8%) and furosemide (2.5%). Figure 2 presented the clinical effects and recommended management of most frequent pDDI pairs observed in this study.

Association between PICU length of stay and patient clinical condition (vasoactive amines use during PICU hospitalization), number of drugs prescribed (more than ten), and severity of pDDI (major and contraindicated) were statistically significant (p < 0.005) in linear regression analysis (Table 3). Multivariate analysis showed an increase in the length of stay of 9.83 days (95% CI = 3.61–16.05; p = 0.002) for children with major or contraindicated pDDI (Table 4).

**TABLE 3:** Association between the PICU length of stay and sex, age group, clinical condition, number of drugs prescribed, and pDDI observed (*n* = 143, Teaching Hospital PICU, Rio de Janeiro, Brazil).

Variables	Increase of PICU length stay (days) (CI 95%)	*p* value
Sex		
Male	Ref	0.814
Female	0.62 (−0.77; 4.54)	
Age (months)		
0–23	Ref	0.610
≥24	1.35 (−3.88; 6.58)	
Clinical condition (vasoactive amines use)		
No	Ref	0.009
Yes	6.79 (1.73; 11.86)	
Number of drugs prescribed		
2–9	Ref	<0.001
10 or more	11.71 (6.83; 16.60)	
pDDI observed		
None, minor or moderate	Ref	<0.001
Major or contraindicated	10.61 (5.60; 15.62)	

pDDI, potential drug-drug interaction; PICU, pediatric intensive care unit. Linear regression. CI, confidence interval; Ref, reference group.

**TABLE 4:** Multivariate analysis for PICU length stay (*n* = 143; Teaching Hospital PICU, Rio de Janeiro, Brazil).

Variables	Increase of PICU length stay (days) (CI 95%)	*p* value
Sex		
Male	Ref	0.955
Female	−0.14 (−5.18; 4.89)	
Age (months)		
0–23	Ref	0.870
≥24	0.42 (−5.53; 4.69)	
Clinical condition (vasoactive amines use)		
No	Ref	0.621
Yes	1.52 (−4.56; 7.60) *p* = 0.621	
pDDI observed		
None, minor or moderate	Ref	0.002
Major or contraindicated	9.83 (3.61; 16.05)	

pDDI, potential drug-drug interaction; PICU, pediatric intensive care unit. Multiple linear regression. CI, confidence interval; Ref, reference group.

## Discussion

This study pointed out relevant data about the occurrence of pDDIs in hospitalized children. Seventy percent of patients aged three days to 14 years old were exposed at least one pDDIs during PICU stay. There were 284 different types of potential drug interactions prescribed 1,123 occasions. Severe pDDIs (major and contraindicated) showed associated with the PICU length of stay increase.

Profile of children in intensive care unit included in this study (mean age, subtle male predominance, respiratory diseases as the principal cause of hospitalization) confirmed the findings of other national and international researches (Corullón, 2007; Vonbach et al., 2008; Ferreira et al., 2012; Lanetzki et al., 2012; Alves et al., 2014; Medina-Barajas et al., 2020). However, the mean length of hospital stays of children in the ICU (11.3 days) was longer than that observed in other Brazilian hospitals, which ranged from 5.5 to 10.6 days (Corullón, 2007; Molina et al., 2008; Lima and Cassiani, 2009; Ferreira et al., 2012; Alves et al., 2014). The length of stay in intensive care can differ due to clinical and social factors. However, institutional factors (practice patterns of physicians, clinical protocols, the proportion of nurses by patients, availability of intermediary care, for example) are the likely primary cause of much of the variability in PICU length of stay and that need to be better investigated in other studies (Pollack et al., 2018). In Latin America, a pDDI investigation in a PICU from a Mexican tertiary hospital for two months, but they did not assess the length hospital stay (Medina-Barajas et al., 2020).

Antibacterials and analgesic drugs ATC subgroups were the most prevalent in this research, which corresponded to data found by Ferreira et al. (2012) in a PICU in Minas Gerais (Brazil). The median of drugs prescribed 12) in the intensive care unit was also close to that observed in other studies (Carvalho et al., 2003; Ferreira et al., 2012).

High frequency of metamizole prescribing reflects common practice in Brazilian hospitals. About 90% of the inpatients had a painkiller prescribed during their hospitalization. A similar percentage (88%) was observed at a pediatric hospital from Brasília (Meiners and Bergsten-Mendes, 2001). Prescription of ranitidine or omeprazole is related to the stress ulcer prophylaxis protocol adopted by the PICU. In a prospective, cross-sectional, observational study in five PICUs in Porto Alegre, ranitidine was also the most commonly used drug for this prophylaxis (Araujo et al., 2010).

National and international studies on adult critical care units indicated a variation of 44.3%–87.9% in the frequency of pDDIs observed (Hammes et al., 2008; Spriet et al., 2009; Reis and Cassiani, 2011). In pediatrics, analysis of prescriptions in the wards of a Brazilian teaching hospital (excluding the PICU, oncology, and emergency) identified seven pDDIs per patient at average, which was slightly lower than the value found in the exclusive PICU analysis conducted in this study (7.85 ± 0.08) (Martinbiancho et al., 2007). The incidence observed in this study (70%) was similar to findings in PICU of United States children hospitals (75%) and another Brazilian PICU (72%) (Lima and Cassiani, 2009; Dai et al., 2016). Other studies found lower frequencies in Indian (63%), Pakistani (59.4%), Mexican (42%), Chilean (41%) PICUs (Santibáñez et al., 2014; Ismail et al., 2017; Rao et al., 2019; Medina-Barajas et al., 2020). An investigation at a Brazilian neonatal intensive care unit found a pDDI prevalence equal to 51% (Queiroz et al., 2014).

The prevalence of pDDIs with major (56.2%) and moderate (37.8%) severity observed was higher than that obtained in cohort with 498,956 American inpatients under 21 years old from pediatric beds (41% and 28%, respectively) (Feinstein et al., 2015). Some frequent pDDIs pairs observed in this study also were described in Chilean PICU (midazolam and omeprazole), in the American cohort (midazolam and ranitidine, fluconazole and ondansetron). The pair midazolam and fentanyl were reported in both studies (Santibáñez et al., 2014; Feinstein et al., 2015).

Of the 1,123 pDDIs found, the drugs belonging to the nervous system’s anatomical group accounted for 55%. Among these, fentanyl was the most frequent medicine prescribed (10%), followed by midazolam (10%). Among the 284 different pDDIs, phenytoin was the drug relatively more present (5%) in pairs. Phenytoin-related DDIs are mainly related to competition for plasma protein binding since phenytoin binding occurs in a proportion of 90%. Drugs with high plasma protein binding displace phenytoin from its site of action, transiently increasing the free drug fraction. In addition, phenytoin is also an inducer of microsomal enzymes, increasing the activity of these enzymes leading to lower serum levels of other drugs (Micromedex Healthcare Series, 2019).

Studies on pDDIs, with different methodologies and scenarios, also show that the combination of midazolam and fentanyl corresponded to the most frequent pDDI (Lima and Cassiani, 2009; Carvalho et al., 2013). However, in intensive care, this pDDI has less clinical relevance due to continuous multiparametric and multimodal monitorization of the patients, including possible signs of abstinence from weaning.

Although a small number of contraindicated pDDIs have been found (1.2%), the high risk of severe harm to patients should be considered (Feinstein et al., 2015). Fluconazole was the most involved drug in this type of DDI. The potential interaction of this antifungal with ondansetron, methadone, or propafenone may result in cardiac abnormalities due to QT interval prolongation. Continuous electrocardiographic monitoring is recommended. Intensive care unit patients with QT interval prolongation have a longer length of hospital stay and higher mortality compared to ICU patients with normal QT (Beitland et a., 2014). Interactions involving fluconazole have also been highlighted in the literature because of the risk of inhibition of CYP3A, CYP1A2, CYP2C8/9, and CYP2C19 isoenzymes, which are responsible for the biotransformation of other drugs (Fonseca and Secoli, 2008; Alvim et al., 2015).

Regarding the other contraindicated pDDIs, the combination of linezolid and amitriptyline may cause a serotoninergic additive effect. This interaction may result in hyperthermia, hyperreflexia, myoclonus, changes in mental state (Lawrence, 2006). The pDDI between sildenafil and sodium nitroprusside is contraindicated due to the risk of severe hypotension (Micromedex Healthcare Series, 2019).

Excessive polypharmacy (more than ten drugs) is commonly required in patients critically ill, and it is a risk factor for adverse drug reactions and medication errors in children (Smith et al., 2012). According to a study that included 54.549 admissions to 42 pediatric hospitals from the United States, a typical inpatient is exposed to 20 drugs over the PICU (median of three days of length stay) (Dai et al., 2016).

The relationship between the number of drugs prescribed and pDDI occurrence, observed in this study, is known, and both are related to a longer length of stay of adults (Moura et al., 2009; Moura et al., 2011; Reis and Cassiani, 2011; Rodrigues et al., 2017) and children (Santibanez et al., 2014; Ismael et al., 2017; Carrilo-Alarcon et al., 2018; Rao et al., 2019; Medina-Barajas et al., 2020) in intensive care units.

However, our findings pointed out the association of pDDI more severe (major and contraindicated) with the increase of PICU length of stay. Severe pDDIs may have greater clinical relevance, and the pDDIs severity differences are rarely investigated in studies, notably those involving pediatric patients. It is the main contribution of this investigation.

The identification of pDDI, especially those that offer more significant risk to the patient, was one of the strategic actions of clinical pharmacists in the investigated PICU. However, there was physician resistance to adjust the prescription. The observation of increased length of hospital stay for patients with major and contraindicated pDDI may contribute to a change in the team’s practices. We believe these results could be generalized to similar settings.

This research has limitations related to real-world evidence studies (Camm and Fox, 2018) and the fact that the patient's clinical condition is based on a *proxy* variable of case complexity. The occurrence of pDDI laboratory and clinical manifestations also cannot be verified. However, the findings presented may support new observational studies in pediatric patients that relate mainly to the pDDI mechanism to the patients' clinical evolution.

There is also a need to identify, and deprescribe (when possible) medicines that have potential contraindicated or more severe pDDIs in critically ill pediatric patients until new evidence is found to substantiate the risk analysis and the possible benefit of keeping the association of certain medications.

It is suggested 1) to discuss the available pharmacotherapeutic alternatives and the possibility to replace the drug by another of the same pharmacological group for the previous risk and benefit assessment by the healthcare team; 2) to monitor the serum level of drugs that may change in the presence of interactions; and 3) to investigate the correlation between some clinical manifestations and the presence of potential DIs, especially those related to the risk of drug ineffectiveness and QT interval prolongation, given the possible consequences of these events.

## Conclusion

This study identified 284 different potential pDDIs in prescriptions of 70.6% of children from a Brazilian teaching PICU involving mainly drugs for the nervous system and antiinfectives for systemic use. More than 60% of pDDIs were classified as contraindicated or with major severity.

Multiple linear regression analyses suggested the association of more severe pDDIs with an increase of PICU length of stay (almost ten days).

It is believed that the results of this research may further the monitoring and prevention of potential drug-drug interactions related to adverse events in children in intensive care and support the design and conduction of new studies assessing the clinical consequences of drug-drug interactions in pediatric patients.

## Data Availability Statement

The raw data supporting the conclusions of this article will be made available by the authors, without undue reservation.

## Ethics Statement

The studies involving human participants were reviewed and approved by Instituto de Puericultura e Pediatria Martagão Gesteira Research Ethics Committee. Written informed consent from the participants’ legal guardian/next of kin was not required to participate in this study in accordance with the national legislation and the institutional requirements.

## Author Contributions

ECL and LCL conceived and designed the study. BDC and NCFB contributed to the acquisition and the interpretation of data for the work. MTS contributed to the statistical analysis. ECL, BDC, NCFB, AGP, RBS, MTS, and LCL discussed the results. ECL drafted the manuscript. All authors critically revised the work and approved the final manuscript.

## Conflict of Interest

The authors declare that the research was conducted in the absence of any commercial or financial relationships that could be construed as a potential conflict of interest.
